# Doxycycline use and adverse pregnancy or neonatal outcomes: A descriptive study using the United States Food and Drug Administration Adverse Event Reporting System database

**DOI:** 10.1002/hsr2.931

**Published:** 2022-11-14

**Authors:** Sammodavardhana Kaundinnyayana, Ashwin Kamath

**Affiliations:** ^1^ Department of Pharmacology, Nepalese Army Institute of Health Sciences College of Medicine Kathmandu Nepal; ^2^ Department of Pharmacology, Kasturba Medical College, Mangalore Manipal Academy of Higher Education Manipal India

**Keywords:** adverse drug event, congenital abnormalities, doxycycline, pregnancy, rickettsia infections

## Abstract

**Background and Aims:**

Doxycycline is recommended for use in rickettsial diseases. The available evidence regarding its safety for rickettsial infection in pregnancy is limited. Our study aimed to describe the adverse events of doxycycline when used during pregnancy for any indication, in terms of adverse maternal and/or neonatal outcomes, using the United States Food and Drug Administration Adverse Event Reporting System (FAERS).

**Methods:**

We used the OpenVigil software for extracting the safety reports from the United States submitted to the FAERS from 2004 to 2021. We manually reviewed reports of doxycycline use resulting in adverse pregnancy outcomes or congenital anomalies to describe the patient and safety event characteristics.

**Results:**

From 2004 to 2021, 59 individual case safety reports containing preferred terms indicative of drug exposure during pregnancy or drug‐induced adverse fetal outcomes were identified in the FAERS database. Following deduplication and manual review, 20 relevant adverse event reports were obtained. Doxycycline was the suspect medication in 13/20 (65%) reports. The common adverse event terms reported were premature delivery/baby in 6 reports, spontaneous abortion in 6, intrauterine death in 2, and various congenital anomalies in the rest. Fifty percent of the safety reports contained other medications which could have potentially caused the outcome.

**Conclusions:**

The number of reported events in the FAERS database of adverse pregnancy/neonatal outcomes following doxycycline use is small, similar to the numbers reported from large cohort or surveillance studies. Given the presence of concomitant medications that could have contributed to the outcome, there does not seem to be a strong signal of harm, although this needs to be confirmed by surveillance studies.

## 1 BACKGROUND

Rickettsial infections during pregnancy can have an adverse impact on maternal and fetal outcomes. Rickettsiosis is an important differential diagnosis in cases of undifferentiated fever, especially in the South‐Asian countries where it is one of the major causes of non‐malarial febrile illness.^1^ Doxycycline, azithromycin, chloramphenicol, and rifamycins are useful in treating the disease to varying extents, with most studies supporting the use of doxycycline.^2^ Doxycycline is generally contraindicated for use in pregnancy due to concerns of possible musculoskeletal and dental defects in the fetus and hepatotoxicity in the mother.^3^, ^4^ However, drugs other than doxycycline are not useful to treat all rickettsial diseases, such as anaplasmosis and ehrlichiosis, and the patient outcomes also may be comparatively inferior.^5^

The Centers for Disease Control and Prevention recommends the use of doxycycline in pregnancy, although it does acknowledge that, based on the available evidence, a teratogenic risk cannot be completely ruled out.^2^ The Indian Council of Medical Research guidelines on the diagnosis and treatment of rickettsial diseases recommends the use of azithromycin in pregnant women,^6^ given the concerns regarding doxycycline use. The available literature evidence regarding doxycycline use for rickettsial infection in pregnancy and patient outcomes is limited.^7^, ^8^, ^9^, ^10^, ^11^

Spontaneous reporting adverse event databases, such as the United States Food and Drug Administration (FDA) Adverse Event Reporting System (FAERS) and Vigibase by World Health Organization, have been extensively used for detecting potential drug safety signals and hypothesis generation. The FAERS database contains adverse events of drugs and biological products reported to the US FDA by patients, doctors, nurses, other healthcare professionals, lawyers, and pharmaceutical companies. The database is open for access to the public and is an important source to detect potential signals of drug‐related problems. There are no published studies reporting on data regarding doxycycline use during pregnancy and the pregnancy and fetal outcomes using such spontaneous databases. The objective of this study is to describe the adverse events of doxycycline when used during pregnancy for any indication, in terms of adverse maternal and/or neonatal outcomes, using the FAERS.

## 2 METHODS

We performed a retrospective descriptive study of the individual case safety reports (ICSRs) in the United States FAERS database from the first quarter of 2004 to the third quarter of 2021. OpenVigil version 2 (OpenVigil 2.1‐MedDRA‐v24) was used to extract the FAERS data.^12^ OpenVigil uses a drug name mapping logic and any cases with errors in drug naming that cannot be resolved (incomplete cases) are excluded, thus providing a cleaned version of the FAERS data.

The ICSRs included in the study had to contain doxycycline reported as a suspect or concomitant medication and an adverse event term reporting at least one pregnancy and/or neonatal outcome and must have occurred in the United States. The adverse event terms in FAERS are coded using the Medical Dictionary for Regulatory Activities (MedDRA) terminology. However, the ICSRs do not have a data field to indicate whether an event occurred during pregnancy. Hence, to identify ICSRs reporting adverse pregnancy or neonatal outcomes due to drug use in pregnancy, we filtered the FAERS data using the standardized MedDRA query (SMQ) term “Pregnancy and neonatal topics” in OpenVigil; this includes the SMQ narrow terms congenital, familial and genetic disorders; fetal disorders; neonatal disorders; termination of pregnancy and risk of abortion; pregnancy, labor and delivery complications and risk factors (excl. abortions and stillbirth).^13^, ^14^ The ICSRs so obtained were further filtered using the following preferred terms: drug exposure during pregnancy; maternal drugs affecting fetus; maternal exposure during pregnancy; fetal exposure during pregnancy.

A major drawback of the FAERS database is the presence of duplicate case reports, occurring due to the reporting of the same event from different sources. We first identified multiple versions (follow‐ups) of the same report by using the case ID, a unique number assigned to each report with the follow‐up numbers added as a suffix, and then, retained only the latest version of the report. Then, the filtered list of ICSRs was reviewed manually to identify possible duplicates by comparing multiple data fields, such as the age, gender, date the report was received at FDA, drug name, event, and reporter country.^15^ With respect to the pregnancy outcomes, only those reports indicative of premature labor or abortion were retained. Reports containing adverse event terms unlikely to be drug‐induced, such as “abortion induced” and “amniotic cavity infection” were excluded. This study was approved by the institutional ethics committee of Kasturba Medical College, Mangalore (IEC KMC MLR 04‐2022/107).

### 2.1 Statistical analysis

The ICSRs were downloaded from OpenVigil in Microsoft Excel file format (Version 2016) for deduplication and manual review. The list of ICSRs was analyzed to determine the various patient and adverse event characteristics such as age, gender, seriousness of the adverse event, time of onset of adverse event, and the reported event(s) in mother and fetus; the data are presented using descriptive statistics.

## 3 RESULTS

Between 2004 Q1 and 2021 Q3, 220352 ICSRs with an adverse event term belonging to the SMQ “Pregnancy and neonatal topics” were reported to FAERS. Of these, 902 (0.41%) contained doxycycline as a suspect or concomitant medication. Seven hundred and three (0.32%) safety reports were from the United States.

Fifty‐nine (59/703, 8.39%) ICSRs contained the preferred term indicative of drug exposure during pregnancy or drug‐induced adverse fetal outcome; these were screened manually to determine whether the listed adverse event terms, supplemented by other information, if available, were indicative of adverse pregnancy or neonatal outcome due to drug exposure during pregnancy. The reasons for excluding the cases are listed in Table 1. Additionally, 3 ICSRs were excluded as they were considered to be potentially duplicate cases.

**Table 1:** Reasons for excluding individual case safety reports in FAERS from the analysis

1. Listed preferred terms do not indicate adverse pregnancy or neonatal outcome
a. Exposure during pregnancy or fetal exposure during pregnancy as the only listed events
b. Drug dispensing error or wrong drug administered
2. Event listed is ectopic pregnancy—unlikely to be due to drug intake during pregnancy
3. Preferred terms unlikely to indicate drug‐induced event
c. Abortion induced, amniotic cavity infection, cystic fibrosis
4. Indication for use of suspect medication was abortion and the adverse event was abortion (complete or incomplete)
5. Paternal exposure during pregnancy (no terms indicating drug intake by the pregnant woman)
6. Maternal age > 50 years
7. Preferred term(s) indicating congenital anomaly in an individual aged > 1 year and not containing terms indicating drug exposure during pregnancy or fetal drug exposure

Abbreviation: FAERS, Food and Drug Administration Adverse Event Reporting System.

**Figure 1:**
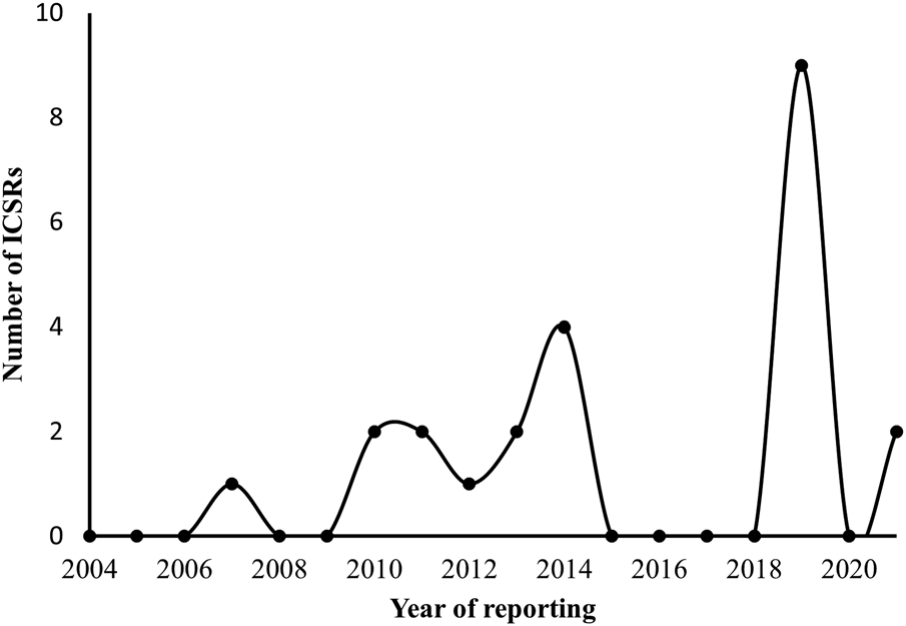
Number of ICSRs of doxycycline‐induced adverse pregnancy or neonatal outcome reported from the United States to FAERS from 2004 to 2021. ICSR, individual case safety report; FAERS, Food and Drug Administration Adverse Event Reporting System; ICSR, individual case safety report.

The final list consisted of 20 (20/703, 2.84%) ICSRs. Maximum reporting occurred in the year 2019; no specific pattern was noted with regard to the yearly reporting of adverse events (Figure 1). In 13 ICSRs (65%), doxycycline was a suspect medication (8 primary and 5 secondary suspect); 14 ICSRs (70%) contained other drugs, in addition to doxycycline, and in 10 (50%) safety reports, the other drugs could have potentially contributed to the outcome as evaluated based on their safety in pregnancy. Eleven ICSRs (47.83%) were reported in adult females; the rest were in newborns or where age was not reported. Table 2 shows the distribution of the adverse event terms as per the SMQ categories.

**Table 2:** Number of ICSRs reporting adverse pregnancy or neonatal outcome to FAERS based on the standardised MedDRA query categories and containing doxycycline as a suspect or concomitant medication

SMQ category (SMQ code)	Number of ICSRs		
	Doxycycline as primary/suspect medication	Doxycycline as concomitant medication	Total
Congenital, familial, and genetic disorders (20000077)	2	3	5
Pregnancy, labor, and delivery complications and risk factors (excl. abortions and stillbirth) (20000186)	4	2	6
Fetal disorders (20000190)	0	0	0
Neonatal disorders (20000191)	2	1	3
Termination of pregnancy and risk of abortion (20000192)	5	1	6

Abbreviations: FAERS, Food and Drug Administration Adverse Event Reporting System; ICSR, individual case safety report; SMQ, standardised MedDRA query.

The principle adverse event terms reported are listed in Table 3. The adverse event outcomes reported were a congenital anomaly in 3 ICSRs (15%), death in 2 (10%), hospitalization in 2 (10%), and others in 13 ICSRs (65%). A brief description of each identified case is presented in Table 4. Five (25%) ICSRs were reported by consumers, and the rest by health professionals.

**Table 3:** Adverse event terms in the ICSRs reporting adverse pregnancy or neonatal outcome to FAERS and containing doxycycline as a suspect or concomitant medication

Doxycycline role	Reported preferred term(s)	Number of ICSRs
Primary suspect	Premature baby	3
	Premature delivery	3
	Abortion spontaneous	2
Secondary suspect	Abortion spontaneous	3
	Conjoined twins/fetal death/heart disease congenital	1
	Craniorachischisis/cerebral ventricle dilatation/conjoined twins/fetal death/neural tube defect	1
Concomitant	Aortic and mitral valve stenosis	1
	Craniosynostosis/scaphocephaly	1
	Intrauterine death	2
	Ankyloglossia congenital	1
	Talipes	1
	Abortion spontaneous	1

Abbreviations: FAERS, Food and Drug Administration Adverse Event Reporting System; ICSR, individual case safety report.

**Table 4:** Details of ICSRs reporting adverse pregnancy or neonatal outcomes to FAERS and containing doxycycline as a suspect or concomitant medication

Patient Age (in years) and Sex	Suspect product active ingredient(s)	Reason for use	Concomitant drug(s)	Reactions	Outcome(s)	Event date	Initial FDA received date
0, Female	Paroxetine	Drug use for unknown indication	Metformin; Doxycycline	Aortic valve stenosis; drug exposure during pregnancy; mitral valve stenosis; multiple congenital abnormalities	Congenital anomaly	January 25, 2005	June 26, 2008
NS, Male	Paroxetine	Product Used For Unknown Indication	Promethazine; Azithromycin; Acetaminophen/Hydrocodone; Doxycycline	Craniosynostosis; drug exposure during pregnancy; scaphocephaly	Congenital anomaly; Hospitalized	‐	November 23, 2010
15, Female	Drospirenone\Ethinyl estradiol	Contraception	Penicillin G; Doxycycline; Sulfamethoxazole\Trimethoprim; Azithromycin; Ceftriaxone; Dexamethasone	Abdominal pain upper; cholecystectomy; cholecystitis chronic; cholelithiasis; chronic tonsillitis; constipation; drug exposure during pregnancy; incision site pain; intrauterine death; nausea; pregnancy; vomiting	Hospitalized; Other outcomes	July 25, 2005	January 14, 2011
29, Female	Isotretinoin	Acne	Insulin; Lisinopril; Simvastatin/Ezetimibe; Doxycycline	Blood alkaline phosphatase increased; blood cholesterol increased; colitis ulcerative; depression; diabetes mellitus inadequate control; diabetic ketoacidosis; drug exposure during pregnancy; gastrointestinal injury/glaucoma; hypertension; hypokalaemia; intrauterine death; irritable bowel syndrome; nephritic syndrome; normochromic normocytic anemia; esophageal ulcer; oesophagitis; pregnancy; sinus bradycardia; suicidal ideation; urinary tract infection	Other outcomes	February, 2005	June 17, 2011
0, Male	Tenofovir disoproxil fumarate	Prophylaxis Against HIV Infection	Doxycycline; Metronidazole; Ibuprofen; Amoxicillin; Acetaminophen; Ferrous sulfate; Folic acid	Ankyloglossia congenital; maternal drugs affecting fetus; post procedural hemorrhage	Congenital anomaly	June 15, 2010	March 08, 2012
31, Female	Mupirocin; Doxycycline	Staphylococcal Infection	‐	Abortion spontaneous; maternal drugs affecting fetus	Other outcomes	July 24, 2012	November 26, 2012
40, Female	Azelaic acid; Doxycycline; Metronidazole	Rosacea	‐	Abortion spontaneous; exposure during pregnancy; pregnancy test positive	Other outcomes	June 04, 2012	June 03, 2013
NS	Potassium chloride; Doxycycline; Oxytocin; Ticarcillin; Clavulanate potassium	‐	‐	Cerebral ventricle dilatation; conjoined twins; craniorachischisis; fetal death; fetal exposure during pregnancy; neural tube defect	Death; Other outcomes	‐	March 07, 2014
NS	Potassium Chloride; Doxycycline; Misoprostol	‐	‐	Conjoined twins; fetal death; fetal exposure during pregnancy; heart disease congenital	Death; Other outcomes	‐	March 07, 2014
0, Male	Fingolimod	Fetal Exposure During Pregnancy	Levetiracetam; Acetaminophen/Hydrocodone; Trazodone; Gabapentin; Phentermine; Lorazepam; Doxycycline	Fetal exposure during pregnancy; gastroesophageal reflux disease; talipes	Other outcomes	September 29, 2012	February 18, 2015
NS	Doxycycline; Betamethasone	Fetal Exposure During Pregnancy	‐	Fetal exposure during pregnancy; premature baby	Other outcomes	‐	May 25, 2019
NS	Doxycycline	Fetal Exposure During Pregnancy	‐	Fetal exposure during pregnancy; premature baby	Other outcomes	‐	May 25, 2019
35, Female	Doxycycline	Product Used For Unknown Indication	‐	Maternal exposure during pregnancy; premature delivery; premature rupture of membranes	Other outcomes	‐	May 27, 2019
25, Female	Doxycycline; Betamethasone	Product Used For Unknown Indication; Maternal Therapy To Enhance Fetal Lung Maturity	‐	Maternal exposure during pregnancy; premature delivery; product use in unapproved indication	Other outcomes	‐	May 27, 2019
20, Female	Doxycycline	Product Used For Unknown Indication	‐	Abdominal pain; abortion spontaneous; maternal exposure during pregnancy; vaginal hemorrhage	Other outcomes	‐	May 28, 2019
NS	Doxycycline	Fetal Exposure During Pregnancy	‐	Fetal exposure during pregnancy; premature baby	Other outcomes	‐	May 30, 2019
25, Female	Doxycycline	Antibiotic Therapy	‐	Live birth; maternal exposure during pregnancy; premature labor; premature rupture of membranes	Hospitalized	‐	June 06, 2019
20, Feamle	Doxycycline	Antibiotic Therapy	‐	Abdominal pain; abortion spontaneous; exposure during pregnancy; vaginal hemorrhage	Other outcomes	‐	June 06, 2019
43, Female	Ceftriaxone; Doxycycline; Azithromycin; Metronidazole; Fluconazole	Pelvic Inflammatory Disease	‐	Abortion spontaneous; exposure during pregnancy	Other outcomes	‐	July 05, 2021
32, Female	Ustekinumab	Psoriasis	Methadone; Betamethasone; Calcipotriene; Clobetasol propionate; Doxycycline; Ketoconazole	Abortion spontaneous; arachnoid cyst; arthralgia; exposure during pregnancy; fatigue; inappropriate schedule of product administration; ovarian cyst; vasodilatation	Other outcomes	June 28, 2021	August 13, 2021

Abbreviations: F, female; FAERS, Food and Drug Administration Adverse Event Reporting System; ICSR, individual case safety report; M, male; NS, not specified.

With regard to safety reports from other countries, 66 ICSRs were available (without manual review); 39 from Canada, United Kingdom 8, Denmark 6, and other countries 13. Eight (12.12%) events were in adult females. Doxycycline was the primary or secondary suspect in 56 (84.85%) safety reports (primary 48 and secondary 8). The outcome was a congenital anomaly in 7 (10.61%), death in 2 (3.03%), hospitalization in 1 (1.52%), life‐threatening event in 1 (1.52%), and other outcomes in 55 (83.33%) ICSRs. The commonly reported events of interest were abortion spontaneous 43, fetal growth restriction 5, lung/respiratory disorder 4, renal aplasia 4, cardiac defect 2, ankyloglossia congenital 2, premature labor 2, 1 each of gastroschisis, polydactyly, trisomy 21, and intrauterine death.

## 4 DISCUSSION

Our study found a limited number of doxycycline‐related safety reports from the United States in the FAERS database. Poor quality or incomplete reports are known issues with the FAERS data. However, based on the available data, half of the reports contained concomitant drugs which could have resulted in adverse pregnancy or neonatal outcomes. Considering the possibility of comorbidities contributing to the outcome, ascribing the outcome to doxycycline use during pregnancy seems difficult. The study findings do not challenge the existing view of cautious use of doxycycline in conditions, such as rickettsiosis in pregnancy, wherein the benefits of drug use likely outweigh the possible small risk of fetal loss or teratogenicity. Moreover, rickettsiosis itself can result in adverse pregnancy outcomes,^7^ further complicating the process of causality assessment.

One of the earliest studies of doxycycline use during pregnancy was in a cohort of 43 pregnant women with mycoplasma infection who received the drug in early pregnancy; a 1‐year follow‐up did not show the presence of any congenital abnormalities in the children.^16^ Studies using data from large registries, such as the Hungarian Case‐Control Surveillance of Congenital Anomalies, which studied the data from 1980 to 1987, found that the use of teratogenic drugs during pregnancy, including doxycycline, was very low, and the risk of congenital anomalies was 0.3%−1.0%.^17^ Another study of the same registry data for the period 1980−1992 found that 0.3% of the 18,515 pregnant women who had offspring with congenital abnormalities had received doxycycline; the rate was significantly higher than that in the control group; however, no significant increased risk was seen in the second and third trimesters.^18^ A retrospective study using a computerized database of 30,049 infants from Tennessee Medicaid born between 1985 and 2000 showed that exposure to doxycycline, among other antibiotics, during pregnancy produced no major increase in the risk of malformations.^19^ Similarly, a review that assessed the use of doxycycline for prophylaxis or treatment of anthrax among pregnant women found only a low risk for congenital anomalies, although the study also highlighted the lack of adequate safety and pharmacokinetic studies in this population.^20^ In contrast, data from the Quebec pregnancy cohort for the period 1998 to 2008 showed a twofold increased risk of circulatory system and cardiac malformations and a threefold increased risk of atrial or ventricular septal defect.^21^ Despite the use of large databases in some of the above‐mentioned studies, there were relatively few exposures to doxycycline in pregnant women, and only a small percentage of these had adverse outcomes. FAERS is one of the largest databases in terms of the number of adverse events reported every year; however, the number of reports identified in our study too was very low. It is also to be noted that the event statistic identified through these studies should be considered along with the background rates of birth defects of 3%−5%.^22^

An alternative to doxycycline for rickettsiosis is azithromycin, which is generally considered safe and preferred for use in pregnancy. Interestingly, a multinational observational study of pregnancy outcomes among women who received macrolides during the first trimester of pregnancy compared with those who received known non‐teratogenic drugs found a trend towards a higher rate of major congenital or cardiac malformations.^23^ A subsequent review of pooled data from observational studies showed that the use of azithromycin is linked to increased odds of spontaneous abortion^24^ as well as major malformations involving the gastrointestinal and musculoskeletal systems.^25^ To add further, a review of published literature on azithromycin use for murine or scrub typhus during pregnancy failed to find benefits in terms of better neonatal outcomes.^7^

Our study has limitations. Besides possible under‐reporting of cases and incomplete/poor quality data in FAERS, the absence of a clear identifier for pregnancy‐related events and the lack of adequate data on drug exposure dates makes ascertaining the temporal relationship difficult. Also, the absence of data on comorbidities makes it difficult to ascertain the true cause of the adverse events. Data on the dose of doxycycline used or the treatment duration was not available. We excluded a few potential duplicate cases based on substantial matching of data in the reports; however, given the absence of a narrative text description, it is difficult to confirm whether these were reports of the same event from different sources or two similar events occurring on the same day.

## 5 CONCLUSIONS

Our study showed that the FAERS contains a limited number of reports of adverse pregnancy or neonatal outcomes due to doxycycline use in pregnancy, similar to the numbers reported from large cohort or surveillance studies; the data is inadequate to support or refute the safety of antenatal use of doxycycline. Even in the available safety reports and the limited data contained therein, half of the reports suggested the presence of potential alternative drugs/causes for the adverse outcome. Hence, the findings seem to be in line with the earlier literature which suggests that the risk of adverse pregnancy or neonatal outcomes is small, at the most, and unlikely to contradict its use in conditions like rickettsiosis in pregnancy. However, continued surveillance by systematic data collection using pregnancy or congenital defect registries is essential.

## AUTHOR CONTRIBUTIONS

**Sammodavardhana Kaundinnyayana**: Conceptualization; data curation; formal analysis; methodology; writing – original draft; writing – review and editing. **Ashwin Kamath**: Conceptualization; data curation; formal analysis; methodology; writing – original draft; writing – review and editing. Both authors have read and approved the final version of the manuscript.

## CONFLICT OF INTEREST

The authors declare no conflict of interest.

## TRANSPARENCY STATEMENT

The lead authors Sammodavardhana Kaundinnyayana and Ashwin Kamath affirm that this manuscript is an honest, accurate, and transparent account of the study being reported; that no important aspects of the study have been omitted; and that any discrepancies from the study as planned (and, if relevant, registered) have been explained.

## Data Availability

The data that support the findings of this study are available in the United States Food and Drug Administration Adverse Event Reporting System database at https://fis.fda.gov/extensions/FPD-QDE-FAERS/FPD-QDE-FAERS.html. Ashwin Kamath had full access to all of the data in this study and takes complete responsibility for the integrity of the data and the accuracy of the data analysis.
